# Prevalence and Risk Factors Associated With Acute Kidney Injury After Transcatheter Aortic Valve Replacement at a Tertiary Hospital in Riyadh, Saudi Arabia

**DOI:** 10.7759/cureus.43381

**Published:** 2023-08-12

**Authors:** Fahad S Alhamad, Yazeed S Almohaimeed, Majd H Alhayzan, Mouath A Alturaymi, Khaled Z Almutairi, Abdullah Almuhanna, Sumayyah Alminhali, Elwaleed Elhassan

**Affiliations:** 1 College of Medicine, King Saud Bin Abdulaziz University For Health Sciences, Riyadh, SAU; 2 College of Medicine, King Saud Bin Abdulaziz University for Health Sciences, Riyadh, SAU; 3 College of Nursing, King Saud Bin Abdulaziz University for Health Sciences, Riyadh, SAU; 4 Medicine and Surgery, King Saud Bin Abdulaziz University for Health Sciences, Riyadh, SAU; 5 Department of Nephrology, King Abdulaziz Medical City Riyadh, Riyadh, SAU

**Keywords:** creatinine baseline, chronic kidney disease, saudi arabia, transcatheter aortic valve replacement, acute kidney injury

## Abstract

Background: Despite recent advancements in techniques, peri- and post-procedural complications still pose a significant challenge in the high-risk transcatheter aortic valve replacement (TAVR) patient population. This study aims to investigate and assess the prevalence of acute kidney injury (AKI) following TAVR, and to identify the risk factors associated with its occurrence.

Methods: We conducted the study at King Abdulaziz Medical City, Riyadh, Saudi Arabia from January 2016 to December 2022. We extracted data from electronic medical records. We categorized and compared patients based on their diagnosis of AKI+ following TAVR, or their absence of AKI- after the procedure.

Results: The study included a total of 344 patients who underwent TAVR. The mean age of the patients was 77.8 ± 8.9 years, 61.8% were male, and the average body mass index was 30.5±7.0. In terms of comorbidities, 70.8% of the patients had diabetes mellitus, 80.5% had hypertension, 8.7% had hypothyroidism, 2.0% had hematological disorders, 23.6% had congestive heart disease, 20.4% had cerebrovascular disease, 4.1% had peripheral vascular disease, 7.3% had cancer, and 34.4% had other comorbidities. The prevalence of AKI was 60 (17.50%) following the procedure. Cerebrovascular diseases showed a significant association with AKI (OR= 3.381, 95% CI, 1.65-6.91, p = 0.001). Chronic kidney disease has a significant effect on AKI (OR = 2.56, 95%CI, 1.02-6.39, p = 0.044). The creatinine level on Day 0 has a significant association with AKI (OR = 1.01, 95%CI, 1.006-1.017, p = 0.0001).

Conclusions: These findings highlight the importance of assessing and managing these risk factors (cerebrovascular diseases, chronic kidney disease, and creatinine level on Day 0) in TAVR patients to mitigate the occurrence and severity of AKI. By understanding and addressing these factors, healthcare providers can potentially improve patient outcomes and reduce the incidence of AKI-associated TAVR procedures.

## Introduction

Transcatheter aortic valve replacement (TAVR) has emerged as a viable alternative procedure for treating individuals with severe aortic stenosis who are deemed unsuitable for surgery [1]. TAVR has exhibited favorable outcomes in both the short and medium term [2]. The diseased aortic valve is replaced with bioprosthetic valves using catheters, either through the transfemoral route [2] or the transapical route [3]. Collectively, the data indicates that the minimally invasive transapical approach is a viable option for patients who are not suitable candidates for open heart surgery due to infeasibility or high risk [4]. TAVR, in comparison to surgical valve replacement, has the potential to lower the risk of atrial fibrillation, bleeding, and acute kidney injury (AKI) [5].

Despite continuous improvements in experience and techniques in recent years, peri- and post-procedural complications still pose a significant challenge in high-risk TAVR patient populations. These complications substantially impact the overall outcome and long-term survival of patients [6]. Specifically, patients selected for TAVR are older and exhibit a higher prevalence of preexisting chronic kidney disease compared to patients selected for surgical aortic valve replacement [7]. Moreover, it should be noted that this technique still requires the use of fluoroscopy and angiography with contrast agents to assist in the proper positioning of the valve [3]. This procedure carries the potential risk of contrast-induced nephropathy (CIN). It is important to note that CIN is associated with a risk of lengthier hospital stays and increased levels of morbidity and mortality [8].

AKI is characterized by the sudden and rapid decline in kidney function, which occurs within a short span of hours or days. This decline leads to the disruption of proper regulation of fluid volume and electrolyte balance, as well as the accumulation of metabolic waste products within the body [9]. The occurrence of AKI following cardiac surgery serves as an independent predictor of both short-term and long-term mortality [10-11]. Indeed, AKI is a common complication observed after TAVR, with reported incidence rates ranging from 8.3% to 58% according to various studies [12-14]. The variations in reported rates may be attributed, at least in part, to the utilization of different definitions for AKI in these studies. A systematic review has shown that TAVR, when compared to surgical aortic valve replacement, is linked to a lower incidence of AKI. However, despite this finding, the occurrence rate of AKI following TAVR remains substantial [7].

While prior studies have identified the clinical and surgical factors linked to an elevated risk of AKI following cardiac surgery, such as the duration of cardiopulmonary bypass (CPB) [15], severe hemodilution during CPB as indicated by nadir hematocrit [16], and low oxygen delivery during CPB, these risks are mitigated in TAVR as CPB is not utilized. However, it is important to note that other potential factors, including older age, diabetes mellitus, and congestive heart failure, still remain contributing factors to AKI following TAVR [15-18]. In addition to the factors mentioned earlier, there are additional potential risk factors for AKI following TAVR. These include the systematic occurrence of short periods of extreme hypotension during rapid pacing for balloon valvuloplasty and valve deployment, as well as the manipulation of large catheters in the aorta of patients who have a high prevalence of diffuse atherosclerosis, which increases the risk of embolization. These factors should be considered when estimating the potential risk of TAVR and its impact on perioperative morbidity and mortality [12]. Another explanation is that the acute reduction of the renal functional reserve can occur due to inflammation and microemboli, which can lead to hemodynamic instability and subsequent dysfunction of various organs, including the kidneys [19]. The available literature on the risk factors associated with AKI following TAVR is limited. This study aims to investigate and assess the prevalence of AKI following TAVR, as well as to identify the risk factors associated with its occurrence.

## Materials and methods

Study setting and study duration

The study was conducted at King Abdulaziz Medical City, Riyadh, Saudi Arabia from January 2016 to December 2022.

Sample size calculation

Based on the specified parameters (alpha error of 0.05, power of 0.95, effect size of 0.1, and an anticipated incidence of AKI following TAVR at 28% [20-21]), the minimum required sample size to determine the occurrence of AKI after TAVR is 287. To account for incomplete and inconsistent data (estimated at 20%), the sample size was increased to 340.

Sampling technique

Since 2016, approximately 15,000 patients have undergone TAVR. For this study, a systematic random sampling technique was employed to retrieve the records of 344 patients. The sampling interval was determined as 43 (calculated by dividing the total population of 15,000 by the desired sample size of 344). The first case was selected randomly, and subsequently, every 43rd case in the sequence was included in the sample until the desired sample size was achieved

Study design and study population 

This study was a retrospective cohort investigation conducted at a tertiary referral hospital, focusing on a single center. The study included all adult patients aged 18 years and older who underwent TAVR for the treatment of aortic stenosis. Patients who met any of the following criteria were excluded from the analysis: (1) previous treatment with renal replacement therapy before undergoing TAVR (received any dialysis modalities within 14 days prior to the TAVR), (2) patients who did not provide research authorization (3) incomplete data regarding the volume of radiographic contrast media administered prior to or during the TAVR procedure, (4) death occurring within seven days of TAVR without a diagnosis of AKI, or (5) missing follow-up data on AKI.

Data collection

Data collection for this study involved extracting various patient-related information from electronic medical records. The collected patient data included: sociodemographic characteristics (age, gender, body mass index (BMI), comorbidities such as diabetes mellitus (DM), hypertension (HTN), hypothyroidism, hematological disorders, cerebrovascular disease (CVD), and peripheral vascular disease (PVD). Regarding the surgical aspect, the following data were recorded: indications or diagnosis for surgery, age of the patients at the time of surgery, and complications encountered during or after the surgical procedure. Lastly, the hospital course data studied AKI and elevated levels of creatinine (measured baseline, daily till the end of the first week). By the Kidney Disease Improving Global Outcomes (KDIGO) definition, AKI is diagnosed by an absolute increase in sCr, at least 0.3 mg/dL (26.5 μmol/L) within 48 hours or by a 50% increase in sCr from baseline within seven days, or a urine volume of less than 0.5 mL/kg/h for at least six hours.

Ethics

The study adhered to the ethical guidelines outlined in the Declaration of Helsinki. It obtained approval from the Ethical Review Board at the relevant institution, and all participating patients provided written informed consent prior to their involvement in the intervention. The study was approved by King Abdullah International Medical Research Center (IRB 23R/146/03).

Statistical analysis 

The patients in the study were categorized and compared based on their diagnosis of AKI+, following TAVR, or their absence of AKI- after the procedure. The two groups, AKI+ and AKI-, were compared to analyze any differences or associations related to the occurrence of AKI following TAVR. Categorical variables were presented as absolute numbers and percentages, and their comparison was conducted using the chi-square test. Continuous data with a normal distribution were reported as mean and standard deviation (SD) and were compared using an independent samples t-test. Continuous variables with a non-normal distribution were reported as medians and interquartile ranges (IQR), and their comparison was performed using the Mann-Whitney U test. To evaluate the predictors of AKI following TAVR, multivariable logistic regression analyses were conducted. The results were reported as odds ratios (ORs) with corresponding 95% confidence intervals (CIs). Initially, univariable associations were assessed for all potential covariates. Covariates with a p-value less than 0.15 in the univariate analysis were entered into the multivariable logistic regression models. Risk variables that demonstrated significance in the univariable analysis were further tested using multivariable modeling. A two-sided p-value of ≤0.05 was considered statistically significant in all analyses.

## Results

The study included a total of 344 patients undergoing TAVR. The mean age of the patients was 77.8 years (range: 47.0-101.0), with a standard deviation of 8.9. Among the patients, 61.8% were male and 38.2% were female. The average body mass index (BMI) was 30.5kg/m2, with a standard deviation of 7.0. In terms of comorbidities, 70.8% of the patients had diabetes mellitus, 80.5% had hypertension, 8.7% had hypothyroidism, 2.0% had hematological disorders, 23.6% had congestive heart disease, 20.4% had cerebrovascular disease, 4.1% had peripheral vascular disease, 7.3% had cancer, and 34.4% had other comorbidities (Table 1).

**Table 1:** Demographic characteristics and comorbidities of patients undergoing transcatheter aortic valve replacement (TAVR) (n= 343)

Variable	Total n (%)
Age mean ± SD	77.8±8.9
Range	47.0-101.0
Sex	
Female	131 (38.2%)
Male	212 (61.8%)
Body mass index	30.5±7.0
Range	15.1-55.5
Diabetes mellitus	243 (70.8%)
Hypertension	276 (80.5%)
Hypothyroidism	30 (8.7%)
Hypothyroidism	7 (2.0%)
Hematological disorders	34 (9.9%)
Congestive heart failure	81 (23.6%)
Cerebrovascular disease	70 (20.4%)
Peripheral vascular disease	14 (4.1%)
Cancer	25 (7.3%)
Chronic kidney disease	41 (12.0)
Others	118 (34.4%)

**Figure 1:**
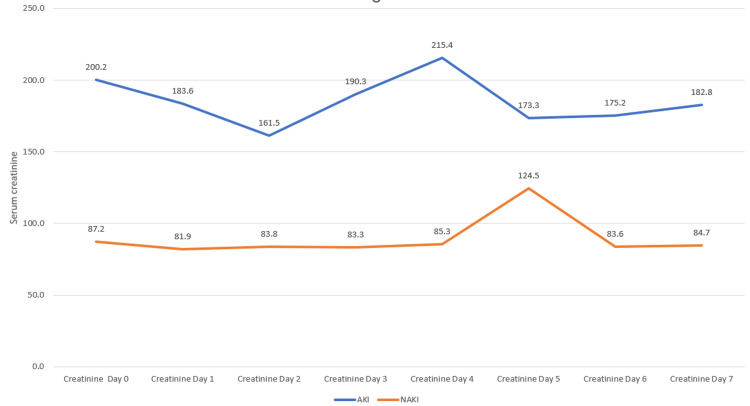
Laboratory measures of creatinine at baseline and daily until Day 7 AKI: Developed acute kidney injury NAKI: Did not develop acute kidney injury

Over the course of the next several days (Days 1-7), the creatinine levels showed some fluctuations in both groups, but the AKI+ group generally maintains higher levels compared to the AKI- group. It is worth nothing that on Day 4, the AKI+ group showed the peak value (215.4 micromol/L) while in Day 5, there is a notable increase in creatinine levels for the AKI- group (124.5 micromol/L) (Figure 1). 

**Figure 2:**
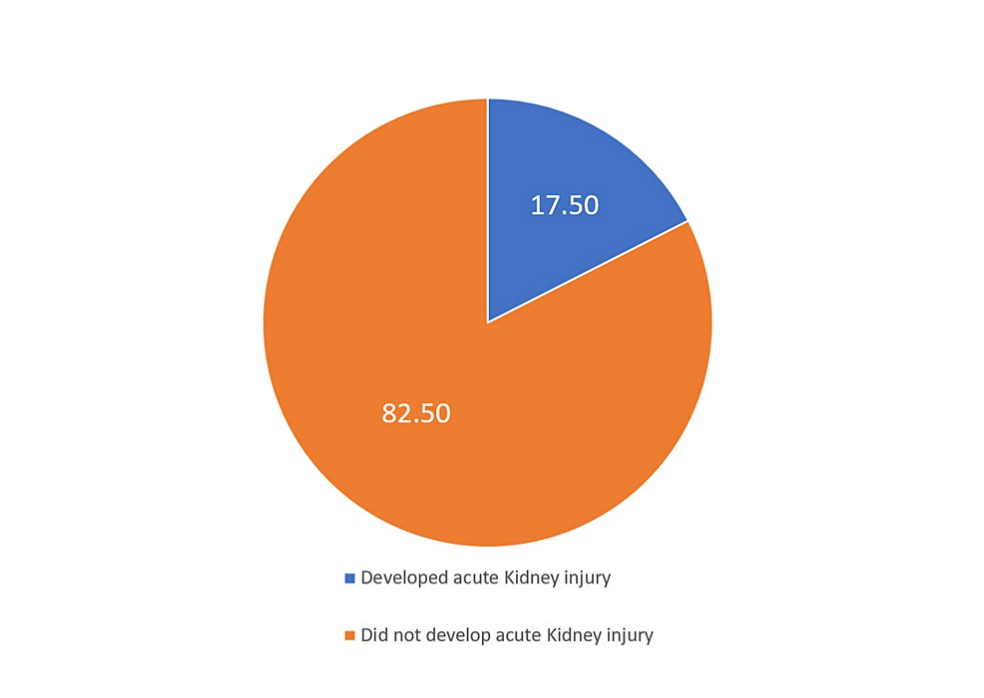
The incidence of acute kidney injury following transcatheter aortic valve replacement (TVAR)

Among the studied population, 60 (17.50%) developed AKI following the procedure, while 282 (82.50%) did not develop AKI (Figure 2). Regarding age and BMI, there were no statistically significant differences observed between the AKI+ and AKI- groups. Similarly, there were no significant differences in the distribution of sex, diabetes mellitus, hypertension, hypothyroidism, or hyperthyroidism between the two groups. However, the presence of cerebrovascular disease and chronic kidney disease showed statistically significant associations with the occurrence of AKI following TAVR. The AKI+ group had a higher proportion of patients with CVD (68.1% VS 31.9% p <0.0001) and CKD (51.2% vs 48.8%, p <0.0001) compared to the AKI- group. Other variables such as hematological disorder, CHF, peripheral PVD, and cancer did not show significant associations with the development of AKI. Creatinine at the baseline was significantly higher among those who developed AKI compared with those who did not develop AKI [110.0(80.0- 320.25) vs 80.5(67.3-97.75, p <0.001)] (Table 2).

**Table 2:** Comparison of studied variables between AKI+ and AKI- groups in patients undergoing transcatheter aortic valve replacement (TAVR) AKI: Acute kidney injury

Studied variables		AKI	No AKI	test statistics	P value
Age	Mean ±SD	76.5±8.3	78.1±9.0	-1.3	0.219
BMI	Mean ±SD	30.8±6.9	30.4±6.9	0.372	0.71
Sex	Male	34(16.0)	178(84.0)	0.88	0.35
	Female	26(20.0)	104(80.0)		
Diabetes mellitus	Yes	43(17.8%)	199(82.2%)	0.29	0.865
	No	17(17.0)	83(83.0)		
Hypertension	Yes	51(18.5)	225(81.5)	0.86	0.353
	No	9(13.6)	57(86.4)		
Hypothyroidism	Yes	4(14.3)	26(86.7)	0.40	0.526
	No	56(17.9)	256(82.1)		
Hyperthyroidism	Yes	1(14.3)	6(85.7)	0.052	1.00^(f)^
	No	59(17.6)	276(82.4%)		
Hematological disorder	Yes	10(29.4)	24(70.6)	3.68	0.055
	No	50(16.2)	258(83.8)		
Congestive heart disease	Yes	17(21.3)	63(78.7)	0.992	0.319
	No	43(16.4)	219(83.6)		
Cerebrovascular disease	Yes	22(31.9)	47(68.1)	12.29	<0.0001
	No	38 (13.9)	235(86.1)		
Peripheral vascular disease	Yes	2(14.3)	12(85.7)	0.11	0.743
	No	58(17.7)	270(82.3)		
Chronic kidney disease	Yes	20(48.8)	21(51.2)	31.42	<0.0001
	No	40 (13.3)	261(86.7)		
Cancer	Yes	6(24.0)	19(76.0)	0.77	0.378
	No	54(17.0)	263(83.0)		
Creatinine baseline	Median (IQR)	110.0(80.0- 320.25)	80.5(67.3-97.75)	-5.82	<0.001

The table presents the results of a logistic regression analysis to evaluate the association between various factors and the development of AKI following the TAVR. The constant coefficient has a significant effect (p = 0.035) with an odds ratio (OR) of 4.44, indicating that there is a higher likelihood of developing AKI. Cerebrovascular diseases showed a significant association with AKI (OR= 3.381, 95% CI, 1.65-6.91, p = 0.001). Chronic kidney disease has a significant effect on AKI (OR = 2.56, 95%CI, 1.02-6.39, p = 0.044). The creatinine level on Day 0 has a significant association with AKI (OR = 1.01, 95%CI, 1.006-1.017, p = 0.0001). These results indicate that higher creatinine levels on Day 0, the presence of cerebrovascular disease, and the presence of chronic kidney diseases were associated with an increased risk of developing AKI. On the other hand, age does not have a significant effect on the development of AKI (p = 0.945) with an OR of 1.69. Gender also does not show a significant association with AKI (p = 0.11) with an OR of 0.56. The presence of hematological disorders does not have a significant effect on AKI (p = 0.292) with an OR of 0.59 (95%CI, 0.22-1.57) (Table 3).

**Table 3:** Factors associated with acute kidney injury (AKI) following the procedure OR: odds ratio; Sig.: significant; SE: standard error

		SE	Wald	Sig.	OR	Lower	Upper
Constant		1.67	4.44	0.035	4.44		
Age		0.02	0.01	0.945	1.69	0.64	4.5
Gender	Male®						
	Female	0.36	2.6	0.11	0.56	0.28	1.13
Hematological disorders	No®						
	Yes	0.5	1.11	0.292	0.59	0.22	1.57
Cerebrovascular diseases	No®						
	Yes	0.37	11.15	0.001	3.381	1.65	6.91
Chronic kidney disease	No®						
	Yes	0.47	4.04	0.044	2.56	1.02	6.39
Creatinine day 0		0	15.1	0.0001	1.01	1.006	1.017

## Discussion

AKI is a significant and frequently observed complication following TAVR. Large registries and meta-analyses have consistently reported an incidence of AKI, at any level, to be approximately 20% [20-21]. This highlights the clinical relevance and importance of monitoring and managing kidney function in patients undergoing TAVR. By understanding the high incidence of AKI, healthcare professionals can proactively implement strategies to mitigate the risk and optimize patient outcomes in this population.

 Our findings revealed that 17.5% of the patients developed AKI. The bivariate analysis demonstrated a significant association between the presence of cerebrovascular disease, chronic kidney disease, and baseline creatinine levels with the development of AKI. Subsequently, through multivariate analysis, these predictors retained their significant associations with AKI.

Similar to our study, Sudarsky et al. [22] conducted research to determine the incidence of AKI following TAVR. They reported an incidence of 18.1%, which is consistent with our findings. Furthermore, Sudarsky et al. identified glomerular filtration rate as the sole significant predictor of AKI in their study. A lower incidence of AKI was reported by Julien et al. [23] in a study conducted on patients who underwent TAVR in the United States between January 1, 2016, and June 30, 2018, a total of 107,814 study patients were included. Among these patients, 11,566 (10.7%) experienced AKI. Among those who developed AKI, 10,220 (9.5%) experienced stage 1 AKI, 134 (0.1%) experienced stage 2 AKI, and 1,212 (1.1%) experienced stage 3 AKI. A higher incidence of AKI was reported by Saia et al. [14], among 102 consecutive patients who underwent TAVR, AKI occurred in 42 patients (41.7%), with 32.4% classified as stage 1, 4.9% as stage 2, and 3.9% as stage 3. The incidence of AKI varied across different access routes, with rates of 66.7% for transapical procedures, 30.3% for transfemoral procedures, and 50% for trans-subclavian procedures. The sole independent predictor of AKI was the use of transapical access. On the other hand, a lower incidence of AKI was reported by Peillex et al. [24] with AKI and acute kidney injury recovery observed in 8.3% and 15.7% of the 574 patients included, respectively. Out of 804 patients who underwent TAVI, Haase-Fielitz et al. [19] reported an incidence of AKI in 13.8% of cases. AKI was found to be an independent risk factor for in-hospital mortality, with a higher risk for those with AKI and additional complications. Modifiable factors such as infection and red blood cell transfusion were associated with a higher likelihood of complicated AKI [19].

Similar predictors were assessed in a study conducted by Abbas et al., [25]. They used the national inpatient sample database, from 2015 to 2018. The study included a total of 173,760 TAVR patients, among whom 20,045 (11.5%) developed AKI. The predictors associated with AKI included chronic kidney disease, weight loss, liver disease, congestive heart failure, cerebrovascular disease, chronic obstructive pulmonary disease, metastatic cancer, and peripheral vascular disease. In the current study, we found that pre-existing chronic kidney disease had the highest odds ratio for developing AKI, OR= 3.381, 95%CI,1.65-6.91. However, Breglia et al. [26] aimed to investigate the occurrence of clinical contrast-induced AKI and subclinical contrast-induced AKI after administering Iodixanol and Iopamidol intra-arterially to patients with an estimated glomerular filtration rate of 60 mL/min/1.73 m² or higher. The findings indicated that the administration of modern contrast media intra-arterially could potentially have a nephrotoxic effect in individuals without pre-existing chronic kidney disease [26].

Limitations

This study has several limitations that should be acknowledged. First, it is based on data collected from a single center, which may limit the generalizability of the findings to other settings or populations. The results may be influenced by specific institutional practices, patient characteristics, and resource availability at the study center. Additionally, the retrospective nature of the analysis introduces inherent limitations, such as the reliance on available medical records and the potential for missing or incomplete data. Additionally, in our study, we encountered limitations in assessing novel risk markers that have been associated with the development of AKI following TAVR. Specifically, we were unable to evaluate the influence of factors such as aortic atheroma volume, which has been linked to AKI, and renal artery stenosis, which has also been suggested as a potential risk marker. Despite the limitations mentioned, we believe that our study makes a valuable contribution to the existing literature. Our findings provide important insights into the incidence of AKI following TAVR and identify associated factors.

## Conclusions

Approximately 20% of patients undergoing TAVR experience the development of AKI. Our study identified several key factors associated with these complications, including pre-existing chronic kidney conditions, baseline creatinine levels, and the presence of cerebrovascular disease. These findings highlight the importance of assessing and managing these risk factors in TAVR patients to mitigate the occurrence and severity of AKI. By understanding and addressing these factors, healthcare providers can potentially improve patient outcomes and reduce the incidence of AKI following TAVR procedures.
